# Granulomatous inflammation in inborn errors of immunity

**DOI:** 10.3389/fped.2023.1110115

**Published:** 2023-02-20

**Authors:** Keith A. Sacco, Andrea Gazzin, Luigi D. Notarangelo, Ottavia M. Delmonte

**Affiliations:** ^1^ Department of Pulmonology, Section of Allergy-Immunology, Phoenix Children's Hospital, Phoenix, AZ, United States; ^2^ Laboratory of Clinical Immunology and Microbiology, Immune Deficiency Genetics Section, National Institutes of Health, Bethesda, MD, United States

**Keywords:** granuloma - etiology, combined immune deficiency, GLILD, rubella (MMR) vaccine, coccidiodomycosis, autoinflammatory and autoimmunological diseases

## Abstract

Granulomas have been defined as inflammatory infiltrates formed by recruitment of macrophages and T cells. The three-dimensional spherical structure typically consists of a central core of tissue resident macrophages which may merge into multinucleated giant cells surrounded by T cells at the periphery. Granulomas may be triggered by infectious and non-infectious antigens. Cutaneous and visceral granulomas are common in inborn errors of immunity (IEI), particularly among patients with chronic granulomatous disease (CGD), combined immunodeficiency (CID), and common variable immunodeficiency (CVID). The estimated prevalence of granulomas in IEI ranges from 1%–4%. Infectious agents causing granulomas such Mycobacteria and Coccidioides presenting atypically may be ‘sentinel’ presentations for possible underlying immunodeficiency. Deep sequencing of granulomas in IEI has revealed non-classical antigens such as wild-type and RA27/3 vaccine-strain Rubella virus. Granulomas in IEI are associated with significant morbidity and mortality. The heterogeneity of granuloma presentation in IEI presents challenges for mechanistic approaches to treatment. In this review, we discuss the main infectious triggers for granulomas in IEI and the major forms of IEI presenting with ‘idiopathic’ non-infectious granulomas. We also discuss models to study granulomatous inflammation and the impact of deep-sequencing technology while searching for infectious triggers of granulomatous inflammation. We summarize the overarching goals of management and highlight the therapeutic options reported for specific granuloma presentations in IEI.

## Introduction

Granulomas represent the result of an immune response induced by an encounter between antigen presenting cells (predominantly monocytes and macrophages) and T cells (both CD4 + and CD8+ T cells) (1, 2). In this regard, granulomas offer an opportunity to explore an interface between the innate and adaptive immune system. However, unlike lymph nodes there is no prerequisite anatomic scaffold for granulomas to develop. Classically, granulomas in mammals have been described as having a spherical structure consisting of a central core of tissue resident macrophages which may merge into multinucleated giant cells surrounded by T cells at the periphery (3). In some cases, the central core of the granulomas shows evidence of necrosis (caseating granulomas), while other forms of granulomas do not show this feature (non-caseating granulomas). These two histological types are exemplified by granulomas occurring in the course of Mycobacterial infection and sarcoidosis, respectively (2, 4). Granulomas may be triggered by infectious and non-infectious antigens (5). Despite significant advances in imaging, histopathology, antimicrobials, and immunomodulation, granulomatous inflammation is a cause for morbidity and mortality in both adults and pediatric patients, particularly as described in common variable immunodeficiency (CVID) (6). Inborn errors of immunity (IEIs) provide a unique insight into the pathophysiology of granulomas and immune susceptibility to specific pathogens that trigger granulomatous inflammation. Tuberculous and non-tuberculous Mycobacterial infection (7) and fungal infections (8) presenting with granulomas, for example, may uncover new forms of IEI. Recently patients with disseminated Coccidioidomycosis, a dimorphic fungal infection endemic to the Southwestern United States, have helped to identify novel variants in genes affecting the immune function and inform on fungal pathogenesis (8). Rubella virus (including the attenuated live vaccine strain) is usually well controlled by the immune system in healthy individuals; however, it has been frequently identified in granulomas of patients with various forms of combined immunodeficiency (CID) (9) and has been associated with significant morbidity and mortality in IEIs (10). In a section of this review, we will highlight the current understanding of the role of infectious agents mentioned and how they interface in the development of granulomas in patients with IEIs.

Furthermore, various IEIs present with granulomas of presumed non-infectious origin involving skin and other organs. The overall prevalence of presumed non-infections granulomas in patients with IEI is estimated to be 1 to 4% with the highest prevalence being among CVID, CID, and CGD (11). In a retrospective single center pediatric study from Turkey, among 82 patients with granulomas who underwent immunological evaluation, 62 carried a diagnosis of IEI. CID was the most common diagnosis and hypogammaglobulinemia was present in 50% of the subjects (12). Granulomas have also been described in autoinflammatory diseases and primary atopic disorders (13).

In this review we will discuss updates in understanding IEIs and variants affecting host defense and predisposing to presumably non-infectious granulomas. The focus of the review will center on non-infectious granulomatous inflammation in CVID, CID, CGD and autoinflammatory disorders. We will highlight the role of infectious agents such mycobacteria and Coccidioides as ‘sentinel’ granulomatous infectious to assist with further workup of possible underlying immunodeficiency. Further, we explore the recent data pertaining to the identification of Rubella virus vaccine-strain in IEI granulomas and discuss how this discovery could shed further mechanistic insights into granuloma pathogenesis.

### Granulomatous inflammation in CVID

CVID is a clinical diagnosis characterized by severe and/or recurrent oto-sino-pulmonary infections, low serum immunoglobulins and impaired vaccine responses (14, 15). Non-infectious complications of CVID, more than infectious history are associated with decreased CVID survival (6). Granulomas in lungs, liver, spleen, lymph nodes and skin were identified in 46 CVID patients within a cohort of 473 subjects (9.7%) (6). A systematic review by van Stigt et al. (16) showed that 50% of CVID patients with granulomatous disease displays extrapulmonary granulomatous manifestation. Granulomatous and lymphocytic interstitial lung disease (GLILD) instead occurs in around 20%–30% of CVID patients (17). GLILD is a clinical, radiologic and pathologic entity characterized by lymphocytic infiltration and/or granuloma of the lung whenever other infectious causes have been excluded. Risk factors for GLILD include history of cytopenias, female gender, age between 20 and 50 years, concomitant hypersplenism and polyarthritis (17, 18). It is associated with increased risk for non-Hodgkin's lymphoma (19). Dysregulated B cells, with increased expression of B cell activating factor (BAFF), which upregulates IFN-*γ* signaling, have been reported in CVID with interstitial lung diseases (20). A recent study identified increased numbers of CD14 + CD16- monocytes and memory T cells, and prominent inflammation in peripheral blood of CVID patients with non-infectious complications. Further, the same patients had higher serum levels of IFN-*γ*, IL-6, IL-18, TNF and T-cell activation markers in peripheral blood (21). These abnormalities improved with T-cell-targeted therapy (21). While the study included various CVID patients with non-infectious complications and not only granulomas, it highlighted the role of T cell dysregulation in the pathogenesis of inflammatory manifestations associated with CVID. Screening high-resolution computerized tomogram (HRCT) is recommended for all CVID patients and should be repeated after 4–5 years if initial screening is unremarkable (22). Further, annual spirometry and 6 min-walk test are considered a cost-effective approach to identify patients who may rapidly progress to GLILD. Immunoglobulin replacement should be targeted to reach and maintain a trough IgG level >1000 mg/dl (22, 23). Increasingly, symptomatic patients may be treated with oral glucocorticoids even though the clinical response to this treatment has shown to be poor (24). Glucocorticoid-sparing agents such as weekly Rituximab 375 mg/m^2^ for 4 weeks repeated every 4 months for 3–4 courses (25), azathioprine 1–2 mg/kg/day or mycophenolate mofetil 250–1000 mg twice daily can be also considered (25). With such treatment, remission of extrapulmonary granulomas affecting the skin, liver and lymph nodes has been reported in 86% of patients (16). Anti-TNF-α therapy has been successful to treat extrapulmonary granulomas in CVID (16, 26).

### Combined immunodeficiencies and granulomatous inflammation

The study of combined immunodeficiencies provides mechanistic insights into the pathophysiologic role of adaptive immune cells in granuloma formation (27, 28). Hypomorphic *RAG* mutations, for example, offer an interesting model to understand granulomatous inflammation. The RAG 1/2 heterotetramer is crucial for the *VDJ* recombination process and for the generation of a diverse repertoire of antigen-specific T-and B-cell receptors (28). The severity of the clinical phenotype correlates with residual RAG1/2 catalytic activity and may range from severe combined immunodeficiency (SCID) in null *RAG 1/2* variants with absent T or B lymphocyte to a less severe phenotype with milder infections, autoimmunity, and granulomas (CID-G/AI) typically with a later onset in life (27). The latter group of patients display preservation of circulating T cells (albeit in reduced numbers and with a predominance of memory cells), and often have normal levels of immunoglobulins and variable response to immunization. High throughput sequencing analysis revealed significant abnormalities of TCR beta repertoire, especially in T regulatory cells, and of BCR repertoire, supporting the notion that relatively higher levels of RAG protein function may allow for partial preservation of the diversity of TCR and BCR repertoires, which however are enriched in self-reactive specificities that may be possibly implicated in granuloma pathogenesis (29). Granulomas in RAG deficiency infiltrate the skin, bones and/or internal organs (30, 31) and can lead to significant morbidity and physical disfiguration (32). In a cohort of 85 patients with RAG deficiency, 30 patients had CID-G/AI phenotype with 15/30 displaying granulomas. Of note, most of these patients had concomitant autoimmunity, with autoimmune cytopenias being the most prevalent autoimmune manifestation (33). In another series of 68 patients with CID-G/AI, granulomatous lesions were identified in 35% of patients with the most common location being lungs and skin but also multiple other tissues (liver, spleen, bone marrow, oropharynx, gut, testis, pancreas); usually more than one site was involved in the same individual (34). In contrast with what reported in CVID, patients with RAG deficiency showed to be refractory to glucocorticoids and biologics (i.e., anti-TNF) requiring allogeneic hematopoietic stem cell transplantation (HSCT) which appears to be the only definitive management for granulomatous inflammation in this disease (33, 35).

Skin and visceral granulomas have been described in several other IEIs with predominance of DNA repair defects including ataxia telangiectasia [AT], Artemis deficiency, Nijmegen-breakage syndrome [NBS], PRKCD deficiency (36, 37) and ligase IV deficiency (38–41) (Table 1). Cutaneous non-infectious granulomas have been extensively described in AT (Table 1) (42). Rarely, patients with AT have had granulomas detected in bones and joints (43). Granulomas are typically observed in AT patients with elevated IgM (44). In a cohort of 44 AT patients, those with granulomas had significantly decreased naïve CD8 T cells in peripheral blood (43). A skewed T cell repertoire has also been reported in AT patients with granulomas (45). IVIG, topical and systemic glucocorticoids, tacrolimus, TNF-α inhibitors have been used with variable success however a patient who underwent allogeneic hematopoietic stem cell transplantation (HCT) had complete remission from granulomas (43).

**Table 1:** Well-described inborn errors of immunity (IEIs) presenting with non-infectious granuloma – presentation, immunophenotype and reported therapies.

	Type	Skin granulomas	Non cutaneous granulomas	Age of presentation (approximative)	Immunologic Findings	Treatment (effect)	References
**CVID**	Non-caseating, tuberculoid, necrobiotic granuloma with perineural invasion	Face, lip, buccal mucosa, nose, cheek, limbs, shoulders, trunk, buttocks, hands, feet	Lungs, lymph nodes, liver, spleen and conjunctiva	Childhood - adulthood	Hypogammaglobinemia	IVIG, Anti- TNF-α, systemic glucocorticoids	(Aghamohammadi et al., s.d.; Harp et al., 2015; Nanda et al., 2014; Stigt, A.C., et al. 2020)
**CID-G/AI (RAG1/2 deficiency)**	Sarcoidal, necrotizing vasculitis, pyoderma gangrenosum, palisades granulomatous dermatitis	Diffuse	Lung, soft tissue, liver, spleen, tongue, gut, testis, bone marrow, adenoids, pancreas, lymph nodes, oropharynx, granulomatous-lymphocytic interstitial lung disease.	Infancy - adulthood (2 −40y).	Mainly T cell lymphopenia. Hypogammaglobulinemia.	Systemic Corticosteroid, cyclosporine, infliximab (partial remission), surgery, HSCT (remission)	(Schuetz et al., 2008), (Delmonte et al., 2018), (De Ravin et al., 2010), (Henderson et al., 2013), (Avila et al., 2010), (Walter et al., 2015), (Sharapova et al., 2013), (Patiroglu et al., 2014), (Buchbinder et al., 2015), (Min et al., 2021), (Farmer et al., 2019), (Geier et al., 2020), (Van Horn et al., 2018)
**Artemis deficiency**	Necrotizing granuloma	Extremities, nose.	–	Infancy (5y – 7 y)	Hypogammaglobulinemia, T cell lymphopenia	HSCT	(Baumann et al., 2022; de Jager et al., 2008; IJspeert et al., 2011)
**PRKCD deficiency**	Non-Langerhans cell histiocytosis. epithelioid granulomas	Face and extremities, limb, elbow	Splenic granuloma	Infancy - childhood (6 mo, 9y)	T and B cell lymphopenia, hypogammaglobulinemia.	IVIG, HSCT	(Esenboga et al., 2018; Mathieu et al., 2015)
**Ataxia-telangiectasia**	Palisading, epithelioid, tuberculoid, necrotizing and non-necrotizing granuloma; Necrobiotic granulomatous inflammation (granuloma annulare); granulomatous acne rosacea.	Limbs, face, trunk, buttocks; trauma-prone areas	Synovial (knee, elbow, wrist), lungs, spleen, liver, larynx, bone marrow, bone (tibia)	Newborn - adulthood (0–31 y)	Decreased IgG, IgA and IgE. Normal/increased IgM, marked decreased B cells and naïve T cells. High AFP	IVIG (progression/partial remission/remission); Topical corticosteroid, systemic corticosteroid, tacrolimus, intralesional triamcinolone injections, antibiotics, prednisolone, adalimumab, infliximab, (partial/transient); HSCT (remission), Isotretinoin [for granulomatous acne rosacea] (remission)	(Amirifar et al., 2020; Cantarutti et al., 2015; Chiam et al., 2011; de Jager et al., 2008; Fleck et al., 1986; Folgori et al., 2010; Joshi et al., 1993; Mitra et al., 2005, 2011; Paller et al., 1991; Privette et al., 2014; ŞentÜrk et al., 1998; Shoimer et al., 2016; Szczawińka-Popłonyk et al., 2020; Woelke et al., 2018)
**TAP1/TAP2 deficiency**	Epithelioid granuloma, necrotizing granulomatous skin lesion	Extremities, midface, legs, other parts of the body	Septal perforation and cartilage destruction	Infancy - adulthood (3–26y)	Complete absence of HLA I on CD8 + cells, low CD8+, normal/increased CD19 + and CD56 + .	Corticosteroids, methotrexate, clarithromycin (null/partial improvement), HSCT	(Darazam et al., 2022; Gadola et al., 2008; Konstantinou et al., 2013; Law-Ping-Man et al., 2018; Moins-Teisserenc et al., 1999; Tsilifis et al., 2021)
**Cartilage hair hypoplasia**	Sarcoidal, tuberculoid, epithelioid, histiocytic palisading (necrobiotic) granuloma.	Limb (monomelic), nose, lips, chin, cheeks, scalp, buttocks.	Bone, nasal septum, larynx, lymph node, spleen, Diffuse (fetal) in skeletal muscle, myocardium, pancreas, spleen, bladder, liver, uterus, thyroid, lungs.	Fetal; infancy - adolescence (1y – 13 y)	T cell lymphopenia, Low IgG and IgA, Intermittent neutropenia	Anti- TNF-α, (partial remission), HSCT (remission)	(Crahes et al., 2013; Leclerc-Mercier et al., 2019; McCann et al., 2014; Moshous et al., 2011; Sathishkumar et al., 2018)
**CGD**	Non-caseating granuloma	Diffuse; granulomatous acne	Gastrointestinal tract, lung, eye, testis, bladder	Infancy - adulthood	–	Corticosteroid, isotretinoin, surgery, HSCT	(Dunogué et al., 2017; Magnani et al., 2014)
**Blau Syndrome**	Non-caseating granuloma	Trunk, extremities	Granulomatous uveitis, hepatic and renal granulomatosis, granulomatous arteritis, granulomatous lymphadenitis, Synovia	Childhood - adolescence (3-12 years)	–	Corticosteroid, immunosuppressive agents	(Jabs et al., 1985; Sfriso et al., 2012; Ting et al., s.d.)
**PLAID**	Non-caseating granuloma	Finger, nose, ears, feet.	Soft palate and larynx.	Birth - childhood.	Low switched memory B-cells. low or low-normal NK cells. Low serum IgM, IgA. poor antibody responses to pneumococcal vaccines. Positive ANA,	Spontaneous disappearance is described; cold avoidance, antihistamines, antibiotic prophylaxis and/or immunoglobulin replacement	(Milner, 2015; Ombrello et al., 2012; Shea et al., 2020)

CVID, common variable immunodeficiency; CID-G/AI, combined immunodeficiency with granulomas and/or autoimmunity; CGD, Chronic Granulomatous Disease; HSCT, hematopoietic stem cell transplant; IVIG, Intravenous immunoglobulin; PLAID, PLCG2 associated antibody deficiency and immune dysregulation; TNF-α, tumor necrosis factor alpha; y, years; mo, months.

The recent identification of RA27/3 Rubella virus (RuV) vaccine strain in some patients has led to the inquiry as to whether RuV antigens may play a causative role for granuloma formation in CID (10, 38). It is unclear as to whether the virus triggers granulomatous inflammation or the impaired host defense allows viral persistence in M2 skewed macrophages and neutrophils (38, 46, 47). Infiltrative granulomas have been described also in TAP1 and TAP2 deficiency (48–52). Recently, 2 cases from Iran of TAP 2 deficiency presenting with granulomas for more than 2 decades before the genetic diagnosis were described. Three relatives of the probands that carried the same homozygous mutation had no clinical manifestation of disease supporting variable expressivity and multifactorial pathogenesis of granulomatous formations (50). Out of 17 cases of TAP2 deficiency reported in the literature, 30% manifested with skin granulomas. Immunomodulatory or immunosuppressant medications are not recommended in this disease because they may cause granuloma exacerbation (50). Data regarding HCT in MHC-I deficiency is limited; one subject displayed persistent regression of skin granulomas 15 years after HCT (53). Cartilage hair hypoplasia patients have granulomas among their clinical manifestations as well (41, 54–56). Rubella virus-associated granulomas have also been described in 21 IEI patients having cytotoxicity defects with significant frequency in MUNC13–4 and RAB27A (Griscelli syndrome type 2) deficiency (57). Of note, GLILD has also been described in Griscelli Syndrome type 2 (58). These data suggest that impaired T cell function allows persistence of macrophages to perpetuate granulomatous inflammation.

### Inflammatory granulomas in phagocytic disorders

CGD is the archetypal IEI for granulomatous inflammation (59, 60). The disorder is characterized by defects in NADPH oxidase due to mutations in 6 known genes – *CYBB, CYBA, NCF1, NCF2, NCF4, and CYBC1* (59)**.** Granulomatous lesions are observed in both autosomal recessive forms (61) and in X-linked forms of CGD (62). Most granulomas in CGD are secondary to infections by organisms predisposing to granuloma formation. The infectious susceptibility and natural history of CGD is described extensively in other reviews (59, 60). However, granulomas of presumed non-infectious origin and post-infectious hyperinflammatory granulomatous inflammation are also prevalent in CGD (63). In a cohort of 71 patients with CGD around 10% suffered from post-infectious granulomas in multiple organs (64). Moreover, in a single center study on inflammatory complications of CGD in 98 patients from France, histological analysis showed presence of granulomatous formation (liver, skin, testes, and ocular) in 22 of 44 patients analyzed. This hyperinflammatory state is often associated with chronic colitis (65), granulomatous cystitis (66) and infections including Staphylococcal liver abscesses or Nocardia infection (67, 68). In these cases, systemic glucocorticoids are co-administered with empiric antimicrobials (68). It appears that hyperinflammation in CGD is triggered by an infectious antigen, however it is often perpetuated due to dysregulation in immune function – particularly defective neutrophil apoptosis (69), skewed NF-*κ*B signaling (70), impaired leukotriene B4 and C5a degradation (71), and upregulation of pro-inflammatory cytokines TNF-α, IL-1β, IL-8, IL-17, IL-6 and G-CSF (72–75). Hyperinflammatory foci, including abscesses, lymphadenitis or granulomas often require surgical excision (76).

Granulomas caused by *Mycobacterium tuberculosis* and non-tuberculous mycobacteria (NTM) are significantly prevalent in IEIs due to phagocytic disorders, and T cell signaling disorders including defects in IFN-*γ*/Il-12 signaling (7, 77). In endemic areas where the Bacille Calmette-Guérin (BCG) vaccine, containing live-attenuated *Mycobacterium bovis* bacilli is administered, IEI patients may present with localized granulomatous inflammation termed BCG-itis or disseminated “BCG-osis” (77–79). This may be the first presentation of CGD or SCID and may present challenges in patient management if HSCT is considered since pre-transplant infection and/or inflammation is associated with poor outcomes. Further, granulomatous lesions may only manifest after engraftment and lead to significant morbidity (80, 81).

Very early onset IBD (VEO-IBD) includes a heterogenous group of monogenic IEIs presenting with inflammatory (non-infectious) bowel disease, occasionally with granulomas, prior to age 6 (82). Commercial targeted gene panels for VEO-IBD typically test over 65 genes (83). Readers are referred to comprehensive reviews on monogenic causes of VEO-IBD for further information (83, 84).

### Disorders of autoinflammation and primary atopic disorders

Autoinflammatory diseases encompass disorders of pathogenic inflammation secondary to intrinsic immune pathway hyperactivation (85). Autoinflammatory syndromes due to hyperactivation of the NF-*κ*B signaling pathway may be associated with granuloma formation. These disorders are also typically associated with exaggerated TNF activity. The major disorder associated with granulomas in this category is Blau syndrome due autosomal dominant *NOD2* pathogenic variants (86). *NOD2* variants are also associated with susceptibility to Crohn's disease, characterized by non-caseating granulomas within the gastrointestinal tract (87). Patients classically present within the first decade of life with a combination of granulomatous dermatitis, erythema nodosum, uveitis and polyarticular arthritis (86). Granulomas infiltrating the liver and kidney have been described in Blau Syndrome (88). Recently, a Japanese patient with a pathogenic *NOD2* variant was diagnosed with Blau syndrome following BCG vaccination suggesting that infectious triggers may play a role in granuloma formation of this disease (89). Granulomatous inflammation has also been observed in autosomal dominant RelA haploinsufficiency leading to NF-*κ*B hyperactivation (90). Systemic glucocorticoids are used as an initial treatment however TNF-α inhibitors have shown significant therapeutic benefit in both RelA haploinsufficiency and Blau syndrome (91, 92).

Phospholipase C gamma 2–associated antibody deficiency and immune dysregulation (PLAID) is a disorder of autoinflammation, autoimmunity, immunodeficiency, and a primary atopic disorder (93). Phospholipase C gamma 2 (PLCG2) hydrolyzes phosphatidylinositol-4,5-bisphosphate into diacylglycerol and inositol trisphosphate, triggering calcium release from the endoplasmic reticulum to mediate cell activation (94). Heterozygous pathogenic deletions in the autoinhibitory domain of PLCG2 cause a PLAID phenotype since it leads to constitutive activation of the PLCG2 enzyme (95). Patients present with recurrent sinopulmonary infections, urticaria triggered by evaporative cooling, granulomatous dermatitis, hypogammaglobulinemia and various autoimmune manifestations (93, 96). Cutaneous granulomatous lesions are present in 25% of patients and among these subjects many developed skin lesions on the nose, ears and fingers within the first few days of life. These lesions may spontaneously resolve in the vast majority of patients however sometimes they may persist and lead to tissue damage and destruction of nasal and auricular cartilage (97). Furthermore, in some cases granulomatous dermatitis may have a later onset affect especially cold exposed areas (97). Histological characteristics of granulomas in PLAID are similar to CVID with a core of tissue resident macrophages including multinucleated giant cells surrounded by a lymphocytic infiltrate. In this disease the most likely pathogenetic trigger is the spontaneous activation of neutrophil and monocytes by cold exposure (98). Treatment includes cold avoidance, daily high dose nonsedating antihistamines, antibiotic prophylaxis and/or immunoglobulin replacement (96). The use of anti-inflammatory drugs or immunomodulators has not shown significant clinical efficacy in PLAID (96).

### Rubella-associated granulomatous inflammation: a potential trigger for significant IEI morbidity

As described previously, live-attenuated RuV RA27/3 vaccine strain has been identified in cutaneous and visceral granulomas in IEI patients (99) (Table 2). This finding highlights the importance for thorough antigen screening in tissue biopsy particularly since treatment of granulomas in IEI has classically focused on use of immunosuppression. The RuV antigen and/or RNA has been identified in at least 66 IEI cases -predominantly cases of AT and CID, and defects of cytotoxicity, but much rarer in primary antibody deficiencies (10, 57). Of note, varicella zoster vaccine, mumps and RuV vaccine strain were all identified in granulomas of a patient with late onset hypomorphic RAG2 deficiency (100). The causal role for RuV in granulomas remains to be defined. Recently, both wild-type and vaccine strain RuV has been isolated from cutaneous granulomas of 4 presumed immunocompetent adults (9, 101). **However, laboratory evaluation in these patients did reveal immunologic abnormalities including lower CD8+ T cells, lower T-cell mitogen responses, reversed ratio of CD8 + to CD4+ T cells, and/or low serum immunoglobulins (101).**

**Table 2:** Well-described pathogens identified in IEI granulomas.

Etiology	Common site of presentation	Types of IEI	Common therapy used	References
**Mycobacteria**	Skin, BCG vaccine site, lymph nodes, bone, lung, bowel, liver, adrenal, aorta, kidney, nerve, muscle, testis, pericardium	MSMD (IL-12/IFN-y axis), anti IFN-y autoantibodies, NEMO deficiency, SCID	Depending on the species, a combination of first and second line antitubercular drugs, antibiotics and surgery. (Wi, 2019)	(Abramowsky et al., 1993; Dolezalova, Karolina et al., 2022; O’Connell et al., 2012; Süleyman et al., 2022; Xu et al., 2019)
**Rubella RA27/3 vaccine strain**	Skin (face limbs, diffuse), lung, spleen, kidney, lymph nodes, bone marrow, and liver.	AT, ADA-SCID and CID (predominant). Also observed in CVID, X-SCID, RAG1/2 deficiency, NBS, XLA, DiGeorge Syndrome, CHH, Artemis deficiency, MHC II deficiency, Marden-Walker syndrome, McKusic syndrome, TAP1 deficiency, WHIM syndrome, Coronin 1A deficiency.	Nitazoxanide, local corticosteroid (no improvement); IVIG (moderate improvement), rapamycin, rituximab, infliximab, interleukin-2 (moderate effect); HSCT (remission).	(Browne et al., 2022; Murguia-Favela et al., 2019; Perelygina et al., 2020; Shoimer et al., 2016)
***Coccidioides* spp.**	Disseminated infection	IL-12/IFN-*γ* and STAT3 signaling pathways disregulation; CID due to *CTPS1* biallelic variants. Impaired TNF-α signaling due to *CLECL7A, PLCG2* variants*;* Impaired Hydrogen peroxyde production *due to monoallelic* DUOX1/*DUOXA1* variant	Antymicotic drugs (fluconazole), IVIG.	(Hsu et al., 2022; Krase et al., 2022; Odio et al., 2017)

AT, ataxia-telangiectasia; CID, Combined immunodeficiency; CHH, Cartilage hair hypoplasia; IFN-γ, interferon gamma; IVIG, intravenous immunoglobulin; MHC, Major histocompatibility complex; MSMD, Mendelian Susceptibility to mycobacterial disease; NBS, Nijmegen-Breakage Syndrome; HSCT, hematopoietic stem cell transplant; SCID, Severe combined immunodeficiency; XLA, X-Linked agammaglobulinemia.

Viral genome sequencing has revealed multiple nucleotide and amino acid substitutions in the RA27/3 vaccine strain identified in IEI granulomas. These vaccine strains have been termed immunodeficiency-related vaccine-derived rubella viruses (iVDRVs) (47). In IEI patients iVDRVs persist within M2 macrophages, neutrophils, and epidermal keratinocytes (34). It is thought that long-term iVDRV reservoir stems from neutrophils and macrophages residing in the bone marrow (46, 47). Further natural history and mechanistic studies are required to further characterize whether iVDRV is causing granulomas in IEIs. Impaired CD8 T cell repertoire could be a plausible mechanistic insight since CD8 T cell memory is critical for Rubella virus control, and CD8 T cell repertoire decreases with age (102, 103). To date no significant clinical improvement for RuV-associated granulomas has been derived by pharmacotherapy and HSCT remains the only definitive management if indicated by the clinical severity of the underlying IEI (10).

### *Coccidioides* – an endemic fungus causing granulomatous inflammation increasingly identified in IEI

Coccidioidomycosis, known as Valley Fever, is caused by the pathogenic fungus *Coccidioides*, endemic to the Southwestern United States (104, 105). Symptomatic illness occurs in around 30% of those infected with disseminated disease in <1% (104, 106). Known risk factors for disseminated disease include secondary immunodeficiency due AIDS, chemotherapy, solid organ-and hematopoietic stem cell transplantation, and immunomodulatory biologics (107). Only 14 patients with disseminated coccidioidomycosis (DCM) had been reported in the literature having mutations impairing immune function – 12 within the IL-12/IFN-*γ* and STAT3 signaling pathways (107–109) and a 5-year-old male with CTPS1 deficiency - a disorder of impaired lymphocyte proliferation (110). A recent publication of a largely adult DCM cohort described mutations in *CLECL7A* and *PLCG2* which impaired TNF-α signaling, and heterozygous variants in *DUOX1* and *DUOXA1* which impaired hydrogen peroxide production (8). A query of the USIDNET database containing information on 5,485 IEI patients in the United States identified 10 patients with a history of Coccidioidomycosis (111). Patients with persistent presumed non-infections granulomatous inflammation can go undiagnosed for coccidioidomycosis (112). In IEI and immunocompromised patients, serology and immunofixation has poor sensitivity thus the diagnosis must be ruled out by tissue biopsy (113, 114). Identification of *Coccidiodes* by tissue biopsy is thus of increasing importance in IEI patients within the Southwestern United states particularly in those where the use of immunomodulators as therapeutics is being considered.

### Diagnostic workup and management of granulomas in IEI

A detailed understanding of the etiology, or at least the inflammatory process of an underlying granuloma is key to direct treatment. For this, obtaining a tissue biopsy for histology and culture is imperative. Apart from acid-fast staining for Mycobacteria, and Giemsa staining for fungal etiology, cultures should be prolonged enough to detect possible fastidious organisms. In some cases where biopsy may not be obtained or cultures remain negative, cell-free DNA testing can help identify less common organisms (115) to enable targeted antimicrobial therapy. Antimicrobial target genes have been identified in granulomas of sarcoidosis patients (116). Cytokine gene expression in granulomas can be quantified using techniques such as RNAScope® (117). Complete surgical excision should be considered whenever granulomas may cause anatomic obstruction or if there is insufficient response to pharmacotherapy. Identification of an underlying pathogen warrants guideline-directed antimicrobial therapy. In the case of antimicrobial therapeutics, this is best done in liaison with infectious disease specialists and tailored to the presumed pathogen based on the underlying IEI. Recombinant interferon-gamma (IFN*γ*) has been used to aid pathogen clearance in IEIs mycobacterial disease, CGD and Coccidioidomycosis. In the case of immunomodulation, there is a longstanding knowledge of using systemic glucocorticoids for inflammatory granulomas in CGD (59, 118). Nitazoxanide, an antiparasitic drug with antiviral properties has been used in IEIs with RuV-associated granulomas (119). Use of oral nitazoxanide was associated with decreased Rubella virus antigen or elimination from granulomas; however, this treatment did not translate into clinically meaningful outcomes (38, 119). Several patients with RuV-associated granulomas underwent allogeneic HCT (120) Patients with lower co-morbid disorders had improved outcomes which suggests that early detection and characterization of RuV-associated granulomas has a bearing on patient outcomes (120).

### Perspectives and futures directions

The advancement of minimally invasive surgical procedures has facilitated obtaining tissue biopsy to characterize granulomas in IEI. Further, histopathologic staining and genetic sequencing can help characterize the inflammatory milieux and possible pathogenic triggers for granulomatous inflammation. This has both prognostic and therapeutic implications, particularly since empiric use of immune suppressive agents can worsen underlying latent infections. The granuloma structure has been described in the mammalian superorder *Archonta* (including primates) and *Laurasiatheria* (including carnivores and ungulates) (121). Granulomatous inflammation in preceding animals such as fish does not reflect the complex architecture seen in primates (122). Non-human primates remain a well characterized model of Tuberculosis-induced granulomas however studies are hindered by length of time needed for granuloma formation and requirement of biosafety level 3 laboratories (123). Mouse models of tuberculous granulomas further do not mirror the granuloma architecture seen in humans (124). Inoculation of iVDRV in hypomorphic models of *Rag1*-mutant mice (125) was unsuccessful in eliciting granulomatous inflammation (126). A heterozygous *Stat4*-mutant mouse model shows predisposition to disseminated Coccidioidomycosis however lung histology is characterized by lymphocyte infiltration rather than granulomatous inflammation (109, 127). *In vitro* human granuloma models have been developed to circumvent the difficulties of studying granulomatous inflammation in animal models (128). An *in vitro* human granuloma model may help characterize mechanisms in granuloma formation as has been observed in various IEI from a clinically phenotypic standpoint. IEIs provide a fascinating template to characterize the heterogeneity and kinetics of granulomatous inflammation. Further characterization of granulomas in IEI can facilitate development of diagnostics and targeted therapeutics for more common granulomatous disorders such as sarcoidosis.
